# 17(R)‐resolvin D1 ameliorates bleomycin‐induced pulmonary fibrosis in mice

**DOI:** 10.14814/phy2.12628

**Published:** 2015-12-10

**Authors:** Masakiyo Yatomi, Takeshi Hisada, Tamotsu Ishizuka, Yasuhiko Koga, Akihiro Ono, Yosuke Kamide, Kaori Seki, Haruka Aoki‐Saito, Hiroaki Tsurumaki, Noriaki Sunaga, Kyoichi Kaira, Kunio Dobashi, Masanobu Yamada, Fumikazu Okajima

**Affiliations:** ^1^ Department of Medicine and Molecular ScienceGunma University Graduate School of MedicineMaebashiJapan; ^2^ Laboratory of Signal TransductionInstitute for Molecular and Cellular RegulationGunma UniversityMaebashiJapan; ^3^ Third Department of Internal MedicineFaculty of Medical SciencesUniversity of FukuiYoshida‐gunFukuiJapan; ^4^ Graduate School of Health SciencesGunma UniversityMaebashiJapan

**Keywords:** Docosahexaenoic acid, inflammation, pulmonary fibrosis, resolvin, *ω*‐3 fatty acid

## Abstract

Idiopathic pulmonary fibrosis (IPF) is a destructive inflammatory disease with limited therapeutic options. Inflammation plays an integral role in the development of pulmonary fibrosis. Unresolved inflammatory responses can lead to substantial tissue injury, chronic inflammation, and fibrosis. The resolvins are a family of endogenous *ω*‐3 fatty acid derived‐lipid mediators of inflammation resolution. Resolvin D1 (RvD1) displays potent anti‐inflammatory, pro‐resolving activity, without causing immunosuppression. Its epimer, 17(R)‐resolvin D1 (17(R)‐RvD1), exhibits equivalent functionality to RvD1. In addition, 17(R)‐RvD1 is resistant to rapid inactivation by eicosanoid oxidoreductases. In the present study, we tested the hypothesis that 17(R)‐RvD1 can provide a therapeutic benefit in IPF by reducing inflammation and pulmonary fibrosis, while leaving the normal immune response intact. Mice were exposed to bleomycin (BLM) via micro‐osmotic pump to induce pulmonary fibrosis, and were then treated with 17(R)‐RvD1 or vehicle by intraperitoneal injection. Administration of 17(R)‐RvD1 from the start of BLM treatment attenuated neutrophil alveolar infiltration, lung collagen content, and Interleukin‐1*β* (IL‐1*β*), transforming growth factor‐*β*1 (TGF‐*β*1), connective tissue growth factor (CTGF), and type I collagen mRNA expression, along with subsequent reduction in histologically detectable fibrosis. The 17(R)‐RvD1‐induced infiltration of inflammatory cells was inhibited by an antagonist of lipoxin A4 receptor/formyl peptide receptor 2 (ALX/FPR2). The administration of 17(R)‐RvD1 at the later fibrotic stage also improved the lung failure. These results suggest that 17(R)‐RvD1 attenuates pulmonary fibrosis by promoting the resolution of neutrophilic inflammation and also provides pulmonary restoration. These data highlight the therapeutic potential of 17(R)‐RvD1 in the management of this intractable disease.

## Introduction

Idiopathic pulmonary fibrosis (IPF) is a progressive, devastating, and lethal disease that has a prevalence of 7–10 per 100,000 people worldwide and a mean survival of 3–4 years (Dempsey 2006). Pulmonary fibrosis is a disorder of the interstitial parenchyma that results in the disruption of lung architecture, and that hinders gas exchange. It is the end stage of a wide range of lung inflammatory conditions (Gross and Hunninghake 2001). Although the etiology of pulmonary fibrosis is not yet completely clear, inflammation, oxidative stress, and damage due to deregulated cytokine expression are definitely involved in pathogenesis. Despite the recent increase in awareness of the mediators and the mechanisms involved in the fibrotic process (Wynn 2008), the most common therapeutic strategy still involves the use of corticosteroids, alone or in combination with other immunosuppressive agents, and has little impact on long‐term survival. However, novel agents are currently being tested as alternative therapies. Pirfenidone is a promising therapeutic agent for the treatment of IPF (King et al. 2014). However, the effects of pirfenidone in patients with advanced stage IPF are unknown (Sakamoto et al. 2013). Similarly, nintedanib also has therapeutic potential. Unfortunately, use of nintedanib is frequently associated with adverse events, such as diarrhea (Richeldi et al. 2014). Currently, highly effective therapies with only minimal side effects are not available for pulmonary fibrosis, indicating the need to develop new treatments that promote the resolution of inflammation.

The course of acute inflammation proceeds through three phases: initiation/development, plateau, and resolution; specific pro‐ or anti‐inflammatory mediators take part in each phase (Nowak 2011). The switch from the initiation to the resolution of inflammation occurs at the cellular level (e.g., neutrophil infiltration, apoptosis, and subsequent elimination by macrophages) and at the molecular level (e.g., the switch from pro‐inflammatory to anti‐inflammatory/pro‐resolving mediators) (Ha‐Na and Surh 2012).

Inflammation is a fundamental component of host defense, which specifically evolved to protect and restore tissue homeostasis by removing deleterious agents and associated damaged tissue (Serhan et al. 2007; Sun et al. 2007). However, chronic or excessive inflammation leads to several common respiratory diseases, including asthma. Many factors, some generated locally, and some migrating from a distance, mediate the inflammatory reaction in the region of infection or allergen exposure. In pulmonary fibrosis in particular, activated inflammatory cells, such as neutrophils and macrophages, are thought to accumulate in the lower airways and to release harmful amounts of reactive oxygen species, as well as various cytokines and growth factors that regulate the proliferation, chemotaxis, and activation of alveolar fibroblasts in the alveolar wall. The activated fibroblasts produce increasing amounts of matrix proteins, distorting the normal lung architecture. Hence, pulmonary fibrosis can be stimulated by chronic or excessive inflammation.

As part of restoring tissue homeostasis, resolution of the immune response not only includes dampening of inflammation, but also promotion of immune system regulatory pathways. The process of resolution originates alongside the initiation of the inflammatory response, when biosynthetic pathways are set in motion that later produce counter‐inflammatory, lipid‐based mediators, including resolvins, protectins, and maresins. Resolvin D1 (RvD1) is a member of the resolvin family of lipid mediators that are derived from docosahexaenoic acid (DHA), an *ω*‐3 fatty acid (Serhan et al. 2000, 2002; Hong et al. 2003). Resolvins of both the D and E series exert potent anti‐inflammatory effects, such as inhibiting neutrophil migration and shortening the resolution phase of acute inflammation (Arita et al. 2005a,b; Bannenberg et al. 2005). Resolvin E1 (RvE1) dampens airway inflammation and hyper‐responsiveness in a murine model of asthma (Aoki et al. 2008; Haworth et al. 2008). Likewise, RvD1 and 17(R)‐resolvin D1 (17(R)‐RvD1), which are generated locally in response to inflammatory stimuli, both possess potent anti‐inflammatory activity, and promote resolution of inflammation (Serhan et al. 2002; Hong et al. 2003). Although they are equally efficacious in decreasing total leukocytic infiltration, at a 10 ng dose, 17(R)‐RvD1 is statistically more potent than RvD1 (Sun et al. 2007). Moreover, in contrast with RvD1, 17(R)‐RvD1 resists rapid inactivation by eicosanoid oxidoreductases. The anti‐inflammatory and pro‐resolving properties of these two resolvin molecules have been investigated in a wide range of experimental inflammatory disease models, including peritonitis (Norling et al. 2011; Chiang et al. 2012), obesity (Titos et al. 2011), acute lung injury (Wang et al. 2011a), diabetes (Hellmann et al. 2011), peripheral nerve injury (Huang et al. 2011), microbial sepsis (Chiang et al. 2012), inflammatory pain (Bang et al. 2012), colitis (Bento et al. 2011), arthritis (Lima‐Garcia et al. 2011; Xu and Ji 2011)**,** and asthma (Rogerio et al. 2012), but not in IPF.

Based on the known effects of 17(R)‐RvD1, in this study, we examined whether 17(R)‐RvD1 could exert anti‐inflammatory and anti‐fibrotic effects in BLM‐induced lung fibrosis and revealed some of the underlying mechanisms of observed effects.

## Materials and Methods

### Mice

Female C57BL/6J mice were obtained from the in‐house colony at the Charles River Animal Facility (Tsukuba, Japan). All mice were handled at the Institute of Experimental Animal Research in the Gunma University Graduate School of Medicine, in accordance with Gunma University Animal Care Guidelines. The protocol was approved by the Animal Care and Experimentation Committee of Gunma University (Permit Number: 14‐039). Mice were used in experiments starting at 8–10‐weeks of age.

### Bleomycin treatment

Bleomycin (Nippon Kayaku Co., Tokyo, Japan) is a well‐established agent for inducing pulmonary inflammation and fibrosis. Continuous BLM administration via micro‐osmotic pump over a limited period closely mimics the progressive nature of fibrosis observed in human patients (Gupte et al. 2009; Lee et al. 2014). Therefore, mice were anesthetized by intraperitoneal pentobarbital injection and a micro‐osmotic pump (Alzet 1007D; DURECT Corporation, CA), containing either bleomycin sulfate (BLM; 2.5 mg/body weight) or 100 *μ*L saline (SAL; control), and designed to deliver its contents at 0.5 *μ*L/h over 7 days, was surgically inserted into the mid‐back subcutaneous region of each mouse. The time point of implantation of micro‐osmotic pumps in mice was designated as Day 0. The mice were sacrificed 7, 14, and 28 days after pump implantation.

### Bronchoalveolar lavage fluid (BALF)

After semi‐excision of the trachea, a plastic cannula was inserted, and the airspace was washed thrice with 0.5 mL of 0.9% NaCl with a 1‐mL syringe. This operation was repeated six times in each mouse. BALF was centrifuged at 1500× ***g*** for 15 min, at 4°C. Differential cell counts were performed using cytospin preparations, after slides containing suspended cells were prepared by centrifugation at 500 rpm for 5 min followed by May‐Grünwald‐Giemsa staining (Merck KGaA, Darmstadt, Germany). Three hundred cells were counted under 400‐fold magnification, and the percentage and absolute number of each cell type were calculated.

### Experimental time course

**Figure 1:**
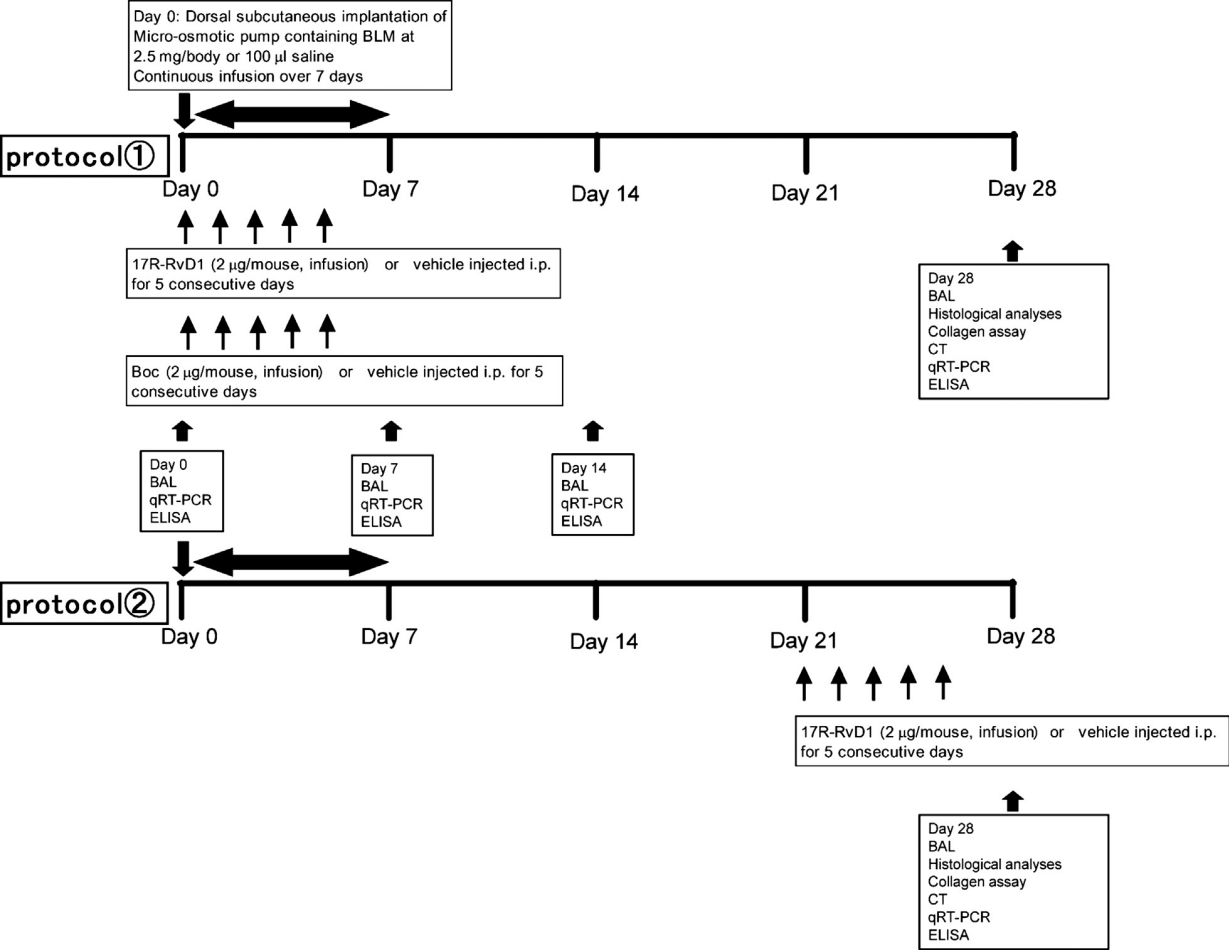
Experimental schedule. Mice were continuously treated with bleomycin (BLM), at 2.5 mg/body weight, or saline control, for 1 week using subcutaneously implanted micro‐osmotic pumps. The time of implantation of the pumps into the mice was designated as day 0. Starting on day 0, mice were injected intraperitoneally (i.p.) with 17(R)‐RvD1 or vehicle (PBS) for five consecutive days (in the first protocol). From day 21, in the latter period of this model, mice were injected intraperitoneally with 17(R)‐RvD1 or vehicle for five consecutive days (in the second protocol). Hence, mice were divided into four groups: saline plus vehicle, saline plus 17(R)‐RvD1, BLM plus vehicle, and BLM plus 17(R)‐RvD1. As part of a time course experiment, on days 0 (pre‐implantation), 7, 14, and 28, mice were sacrificed, and lung samples were collected for further analysis. ELISA was employed to determine hydroxyproline content.

The time course of this study spanned 28 days (Fig. 1). We designed two experimental protocols to examine the effect of 17(R)‐RvD1. At first, 17(R)‐RvD1 was injected to mice from day 0 to day 4, during the inflammatory stage, after BLM treatment in the first protocol. Secondary, 17(R)‐RvD1 was injected to mice from day 21 to day 25, during the fibrotic stage, after BLM treatment in the second protocol. We evaluated cellular changes in BALF and pathological changes in lung tissue after BLM treatment. Pulmonary inflammation was evaluated by measuring total cell count and distribution of immune cell types in BALF, and expression of Interleukin‐1*β* (IL‐1*β*) mRNA in lung tissue. BAL was performed at Day 0 (before BLM or SAL injection) and at days 7, 14, and 28. In addition, histological evaluation was performed at day 28. At days 14 and 28, analyses of gene expression of transforming growth factor‐*β*1 (TGF‐*β*1), tissue growth factor (CTGF), and type I collagen were performed to elucidate the mechanism of fibrotic change in the first protocol. Mice were divided into four groups in each protocol. The first group of mice, designated as the SAL/veh group, were control mice; mice in this group were treated with SAL via micro‐osmotic pump and injected intraperitoneally (i.p.) with PBS as vehicle. The second group of mice, designated as the BLM/veh group, was treated with BLM via micro‐osmotic pump and injected i.p. with vehicle. The third group of mice, designated as the SAL/RvD1 group, was treated with SAL via micro‐osmotic pump and injected i.p. with 17(R)‐RvD1, purchased from Cayman Chemical (Ann Arbor, MI). The final group of mice, designated as the BLM/RvD1 group, was treated with BLM via micro‐osmotic pump and injected i.p. with 17(R)‐RvD1. Mice injected with 17(R)‐RvD1 received 2 *μ*g 17(R)‐RvD1 per mouse, a dose chosen based on our data from a preliminary dosage experiment (data not shown). The 17(R)‐RvD1 was dissolved in alcohol. Just before using 17(R)‐RvD1 on my experiment, we evaporated alcohol and added 100 *μ*L of PBS (for a final concentration of 0.02 *μ*g/*μ*L). After that, we administered it quickly by i.p. injection to mice, each day, for five consecutive days. Similarly, vehicle‐treated mice were injected with 100 *μ*L of PBS per day, for 5 days.

### N‐t‐Boc‐Phe‐Leu‐Phe‐Leu‐Phe (Boc‐PLPLP)

Boc‐PLPLP is a chemotactic peptide antagonist of Formyl Peptide Receptor (FPR) that is the receptor of RvD1 and binds to neutrophils. We purchased it from MP Biomedicals (Solon, OH, USA) and administered 2 *μ*g/body of Boc‐PLPLP to mice from day 0 to day 4, the same period of 17(R)‐RvD1 treatment, to demonstrate the 17(R)‐RvD1's inactivation.

### Histology

Lungs of mice were harvested at 28 days after installation of the micro‐osmotic pumps. Each left lung was fixed in 20% formaldehyde neutral buffer solution (Wako, Osaka, Japan), and embedded in paraffin. Tissue sections were stained with hematoxylin and eosin (H&E) or with Masson's trichrome. The severity of fibrosis was assessed by light microscopy. The Ashcroft scale was used for the quantitative histological analysis of the fibrotic changes induced by BLM. The degree of fibrosis in each lung section was assigned a mean fibrotic severity score from 0 (normal) to 8 (total fibrosis) (Ashcroft et al. 1988). The mean fibrotic severity scores were compared between treatment conditions. The criterion for grading the severity of pulmonary fibrosis included infiltration by inflammatory cells, thickening of alveolar walls, and collagen deposition.

### Determination of hydroxyproline content

After the mice were sacrificed, their lungs were removed, snap frozen, and stored at −80°C. Total hydroxyproline content of the left lung was measured as a proxy for lung collagen content. Hydroxyproline assay was performed as follows. Briefly, lungs were lyophilized for at least 48 h, and pulverized ground lung was mixed with 100 *μ*L of 6N HCl and then incubated for 3 h at 120°C. The resulting acid hydrolysates and standards were applied to an ELISA plate (Corning Incorporated, NY) and dried by evaporation under vacuum. Hydrolysates and standards were incubated at room temperature for 5 min after addition of 100 *μ*L of the Chloramine T reagent (BioVision, Milpitas, CA) to each sample well. After subsequent addition of 100 *μ*L of the p‐dimethylaminobenzaldehyde (DMAB) reagent (BioVision, Milpitas, CA) to each well, samples were incubated for 90 min at 60°C. The absorbance of each sample at 560 nm was then measured using a microplate reader (Molecular Devices, Sunnyvale, CA).

### Computed tomography (CT) image

The previous study has shown that CT image is a reliable quantitative tool to investigate experimental lung fibrosis in mice (De Langhe et al. 2012). To characterize the nature and degree of lung fibrosis after BLM treatment, we performed CT examinations of the mice. The tissue density of the mouse lungs was quantified by CT at day 28, and the extent of lung fibrosis was evaluated in each protocol. An increase in lung density could indicate greater inflammation and fibrosis. We imaged the radiological features of the mouse lungs while mice were anesthetized with isoflurane (Abbott, Tokyo, Japan). In order to optimize the image, we reduced cardiac and respiratory motion.

### Real‐time PCR

Right lungs from mice in each group were pooled, and total RNA was extracted using TRIzol Reagent (Thermo Fisher Scientific, Foster City, CA) according to manufacturer's instructions. Reverse transcription of 1 *μ*g of total RNA, and amplification of the resulting cDNA, was performed using TaqMan Reverse Transcription Reagents (Thermo Fisher Scientific). To exclude amplification of genomic DNA, reactions were also run in the absence of reverse transcriptase. Quantitative reverse transcription PCR (qRT‐PCR) was performed using the Applied Biosystems 7300/7500 Real Time PCR systems (Thermo Fisher Scientific). The cDNAs were amplified in duplicate. Mouse, TGF‐*β*1, CTGF, IL‐1*β*, matrix metalloproteinase 9 (MMP‐9), inhibitor of metalloproteinases‐1 (TIMP‐1) and GAPDH were amplified (with the latter used as an endogenous control for normalization) using the TaqMan Universal PCR Master Mix (Thermo Fisher Scientific) with target gene specific TaqMan Gene Expression primers and 6‐carboxyfluorescein (FAM)‐labeled probes (Thermo Fisher Scientific). Type I collagen and *β*‐actin were amplified using the SYBR Green Real‐time PCR Master Mix (Toyobo, Osaka, Japan) with appropriate primers. Quantitative (real‐time) PCRs were performed in an Optical 96‐well Reaction Plate (Thermo Fisher Scientific). The thermal cycler parameters were 50°C for 2 min, followed by 95°C for 10 min, followed by 40 cycles of 95°C for 15 sec and 60°C for 1 min. Quantitative analysis of gene expression utilized the comparative C_T_ (ΔC_T_) method, in which C_T_ is the threshold cycle number.

### Statistical analysis

Numerical data were expressed as mean ± SEM. Non‐parametric analysis of variance, via the Kruskal–Wallis method, was used to determine the degree of significance of differences between the groups. The Mann–Whitney *U* test was used to test for significant differences between pairs of groups. A value of *P *<* *0.05 was considered significant.

## Results

### The anti‐inflammatory effect of 17(R)‐RvD1 in the BLM mouse model

**Figure 2:**
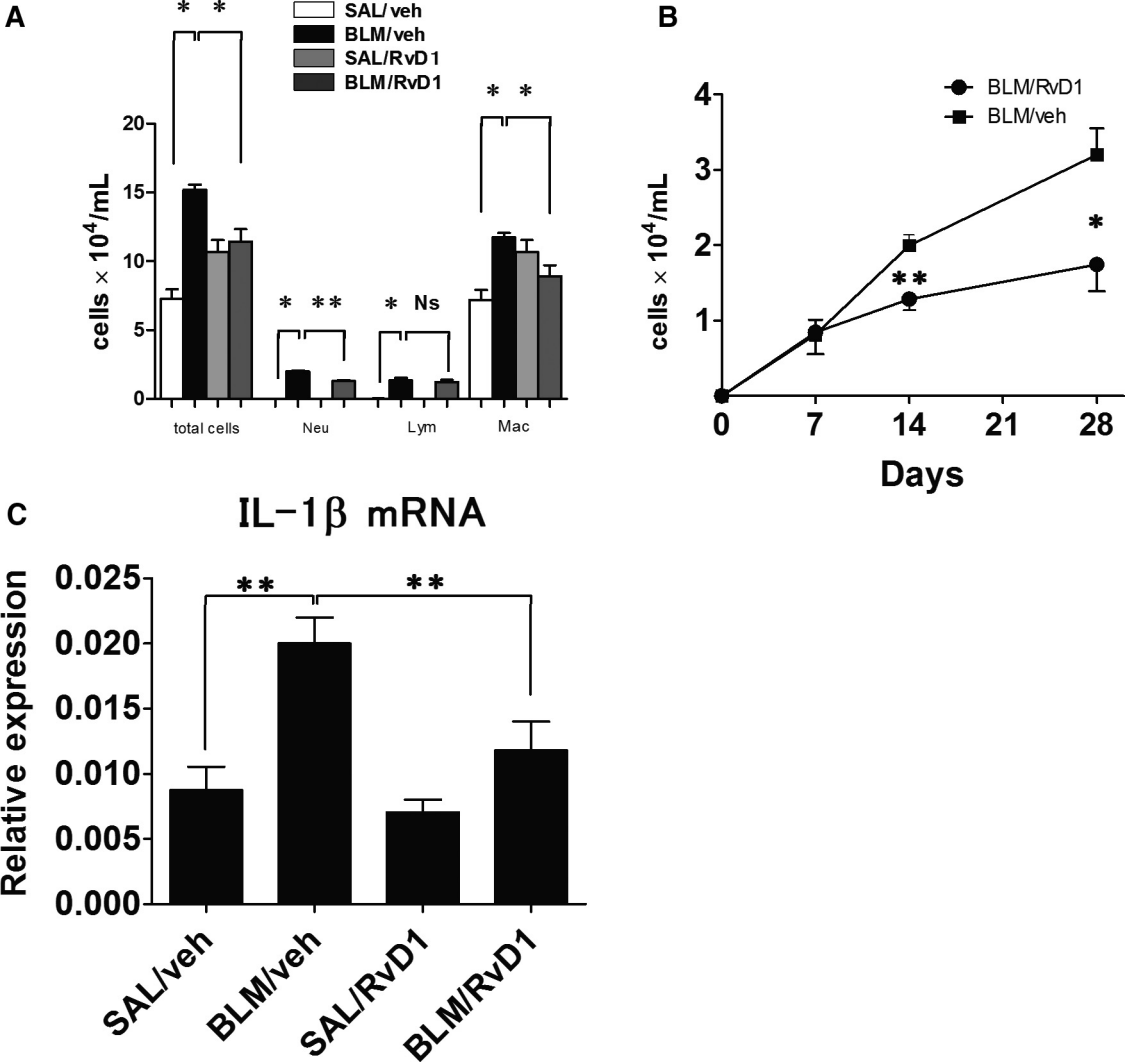
Bleomycin (BLM)‐induced inflammatory cell infiltration and IL‐1*β* mRNA in lung tissue after vehicle (veh) or 17(R)‐RvD1 treatment. Four groups of mice were assayed. SAL/veh mice were treated with saline via micro‐osmotic pump and injected with vehicle intraperitoneally (i.p.), while BLM/veh mice were treated with BLM and injected with vehicle; SAL/RvD1 mice were treated with saline and injected with 17(R)‐RvD1, and BLM/RvD1 mice were treated with BLM and injected with 17(R)‐RvD1. (Vehicle was PBS) (A) Cell counts in BALF obtained from mice in each group at day 14. Data are expressed as means and standard errors of the mean (means ± SEM) of six mice per group (*n* = 6). Neu, neutrophil; Lym, lymphocyte; and Mac, macrophage (B) Changes in numbers of neutrophils in BALF obtained at days 0, 7, 14, and 28 from mice treated with BLM and injected with vehicle or 17(R)‐RvD1. Bars represent means ± SEM of eight mice per group (*n* = 8). (C) Expression of IL‐1*β* mRNA, a potent pro‐inflammatory cytokine. Data are expressed as means ± SEM of eight mice per group (*n* = 8). **P <* *0.01 and *P <* *0.05 denote significant differences in values as determined by the Mann**–**Whitney *U* test.

**Figure 3:**
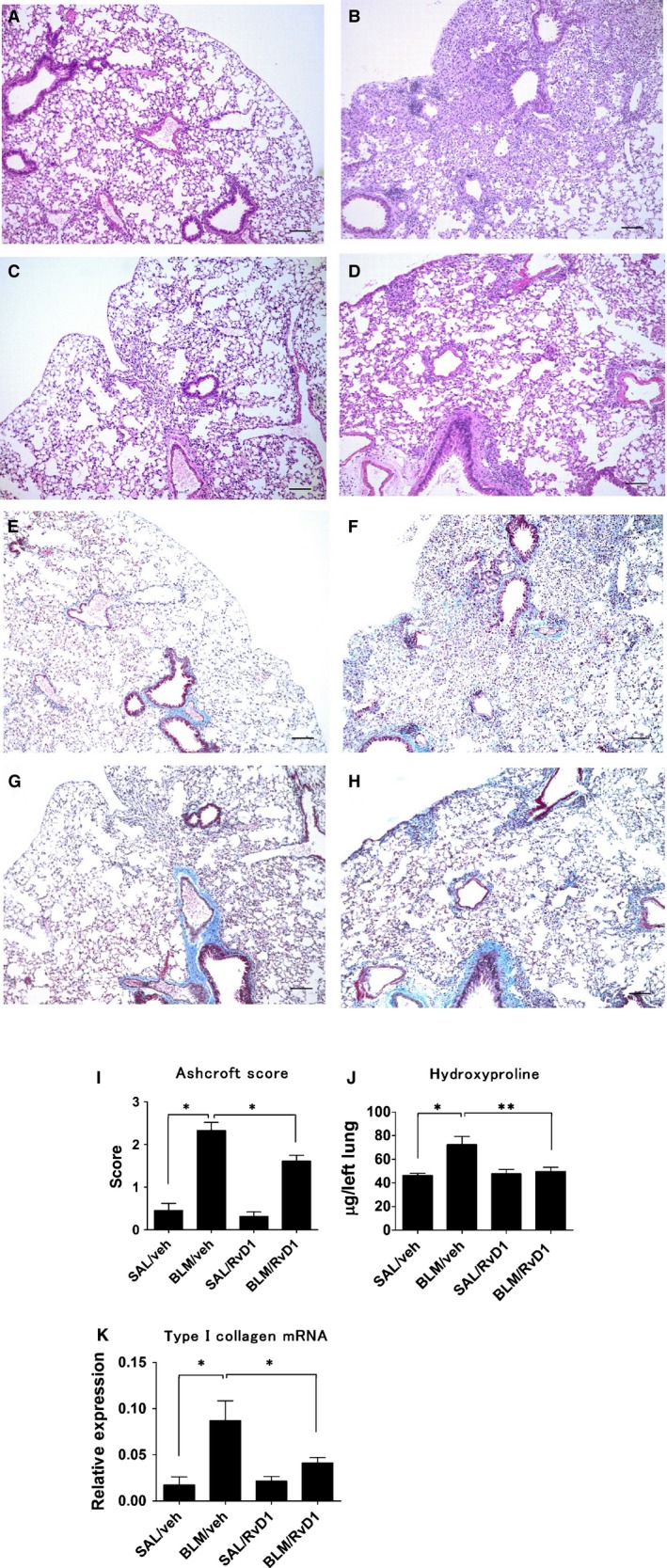
Histrogy of lung tissue. Histology of sections of lung tissue at day 28 from mice treated with bleomycin (BLM) or SAL, and subsequently repeatedly injected with either 17(R)‐RvD1, also abbreviated herein as RvD1, or veh. (A–D) representative light micrographs of lung tissue sections, stained with hematoxylin‐eosin. (A) SAL/veh mice. (B) BLM/veh mice. (C) SAL/RvD1 mice. (D) BLM/RvD1 mice. (E–H) Corresponding with A–D in order, representative light micrographs of lung tissue sections stained with Masson's trichrome to detect collagen content. Scale bars indicate 100 *μ*m. Histopathology shows dense areas of lung fibrosis after treatment with BLM and injection with vehicle (B and F). There was a markedly reduced focal fibrosis in sections from mice treated with BLM and injected with 17(R)‐RvD1 (D and H). Most areas of the lung parenchyma were preserved (afibrotic) in mice treated with saline (A, C, E, and G). (I) Ashcroft fibrosis score of lung sections from mice from each of the four treatment groups. Data are expressed as means ± SEM of eight mice per group (*n* = 8). (J) Total lung collagen levels were measured by hydroxyproline content assay. Data are expressed as means ± SEM of 10 mice per group (*n* = 10). (K) Transcription levels of Type I collagen mRNA were measured by real‐time quantitative RT‐PCR. Data are expressed as means ± SEM of five mice per group (*n* = 5). *P <* *0.05; **P <* *0.01.

**Figure 4:**
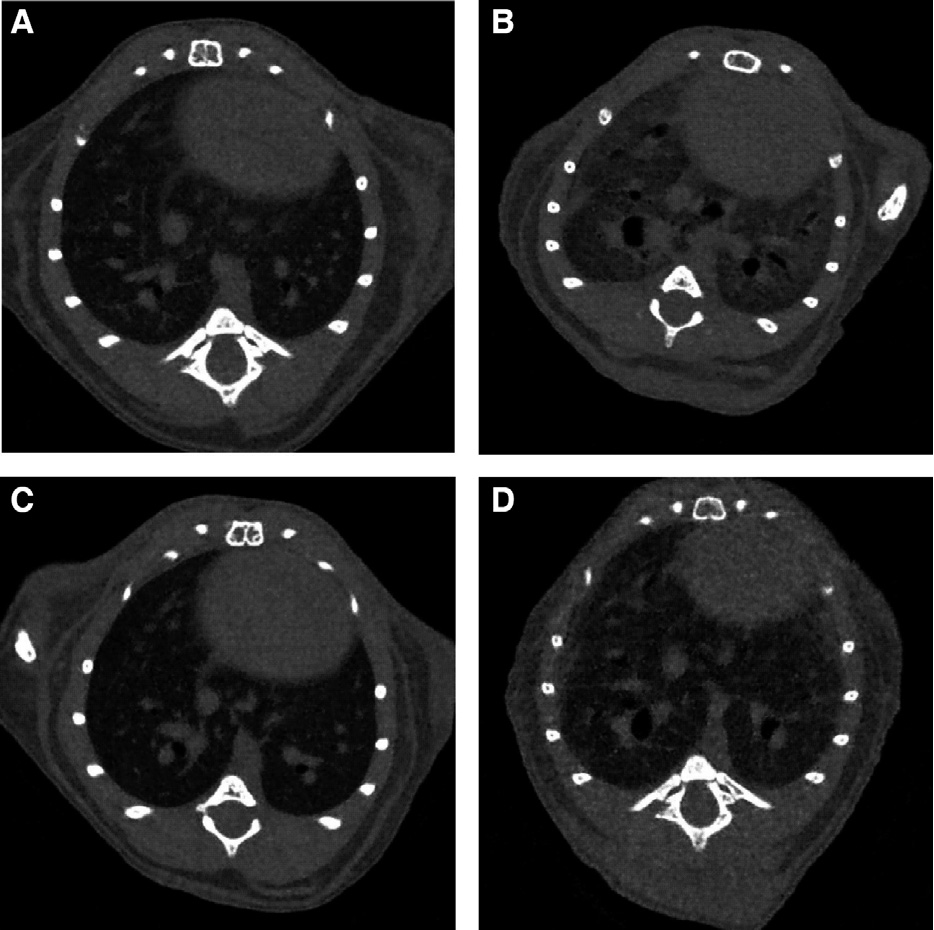
Computed tomography (CT) images of lung tissue. Density of mouse lung, as visualized by CT image at day 28. The increase in lung density may indicate more extensive fibrosis and chronic inflammation. It is noted that the higher density site appears more brighten in this CT image. (A) SAL/veh treatment. (B) Bleomycin (BLM)/veh treatment. (C) SAL/RvD1 treatment. (D) BLM/RvD1 treatment.

**Figure 5:**
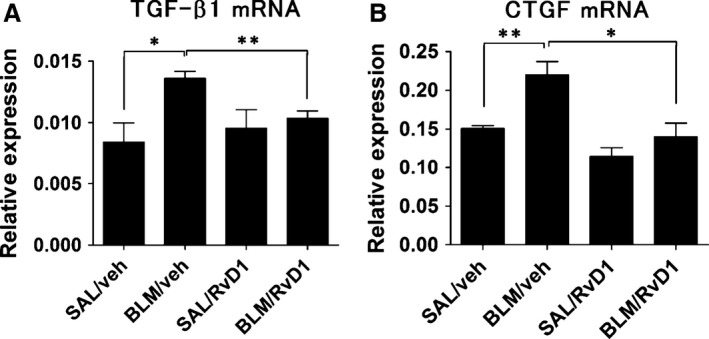
Expression of TGF‐*β* and CTGF mRNA. Gene expression of fibrogenic mediators that are components of the TGF‐*β* signaling pathway at day 14. Relative levels of expression of TGF‐*β*1 (A) and CTGF (B) mRNAs were measured by qRT‐PCR. Data are expressed as means ± SEM of nine mice per group (*n* = 9). *P <* *0.05; **P <* *0.01.

**Figure 6:**
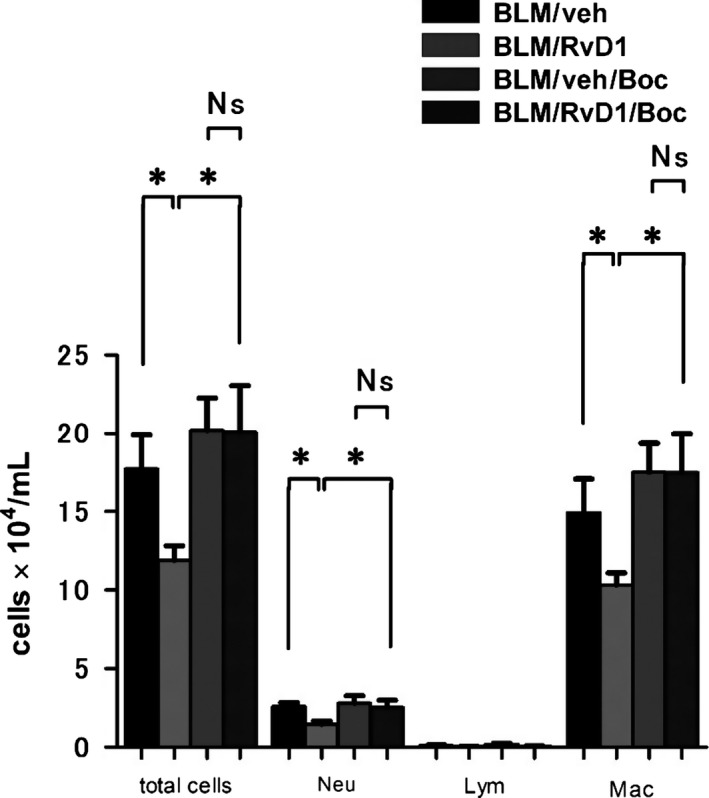
Boc‐PLPLP inhibited the anti‐inflammatory effect of 17(R)‐RvD1 in bleomycin (BLM)‐induced inflammatory cell infiltration. Four groups of mice were assayed. All mice in each group were treated with BLM via osmotic pump. BLM/veh mice were injected with vehicle intraperitoneally (i.p.). BLM/RvD1 mice were injected with 17(R)‐RvD1. BLM/veh/Boc mice were injected with vehicle and Boc‐PLPLP i.p. injection. BLM/RvD1/Boc mice were injected with 17(R)‐RvD1 and Boc‐PLPLP. Cell counts in BALF obtained from mice in each group at day 14. Data are expressed as means ± SEM of five mice per group (*n* = 5). *P < 0.05

**Figure 7:**
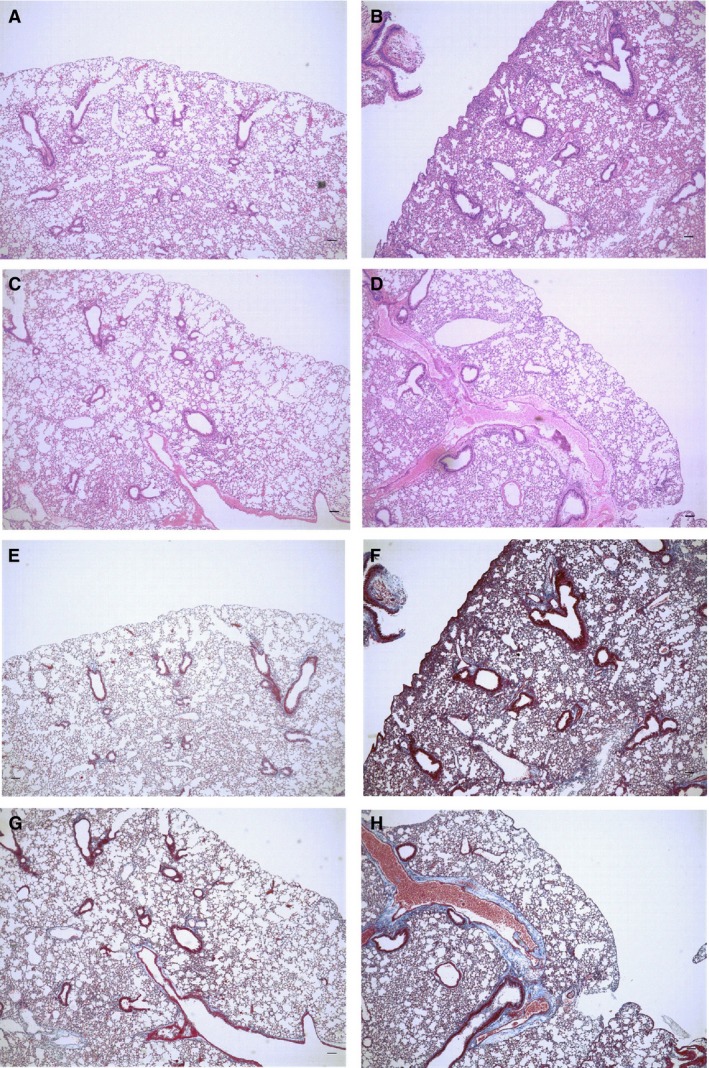
Histology of lung tissue. Histology of sections of lung tissue from mice after treatment of either 17(R)‐RvD1 or vehicle from day 21 to day 25 with bleomycin (BLM) or SAL. (A–D) representative light micrographs of lung tissue sections, stained with hematoxylin‐eosin. (A) SAL/veh mice. (B) BLM/veh mice. (C) SAL/RvD1 mice. (D) BLM/RvD1 mice. (E–H) Corresponding with A–D in order, representative light micrographs of lung tissue sections stained with Masson's trichrome. Scale bars indicate 100 *μ*mol/L. Histopathology shows dense areas of lung fibrosis after treatment with BLM and injection with vehicle (B and F). There was a markedly reduced, focal fibrosis in sections from mice treated with BLM and injected with 17(R)‐RvD1 (D and H). Most areas of the lung parenchyma were normal in mice treated with saline (A, C, E, and G).

**Figure 8:**
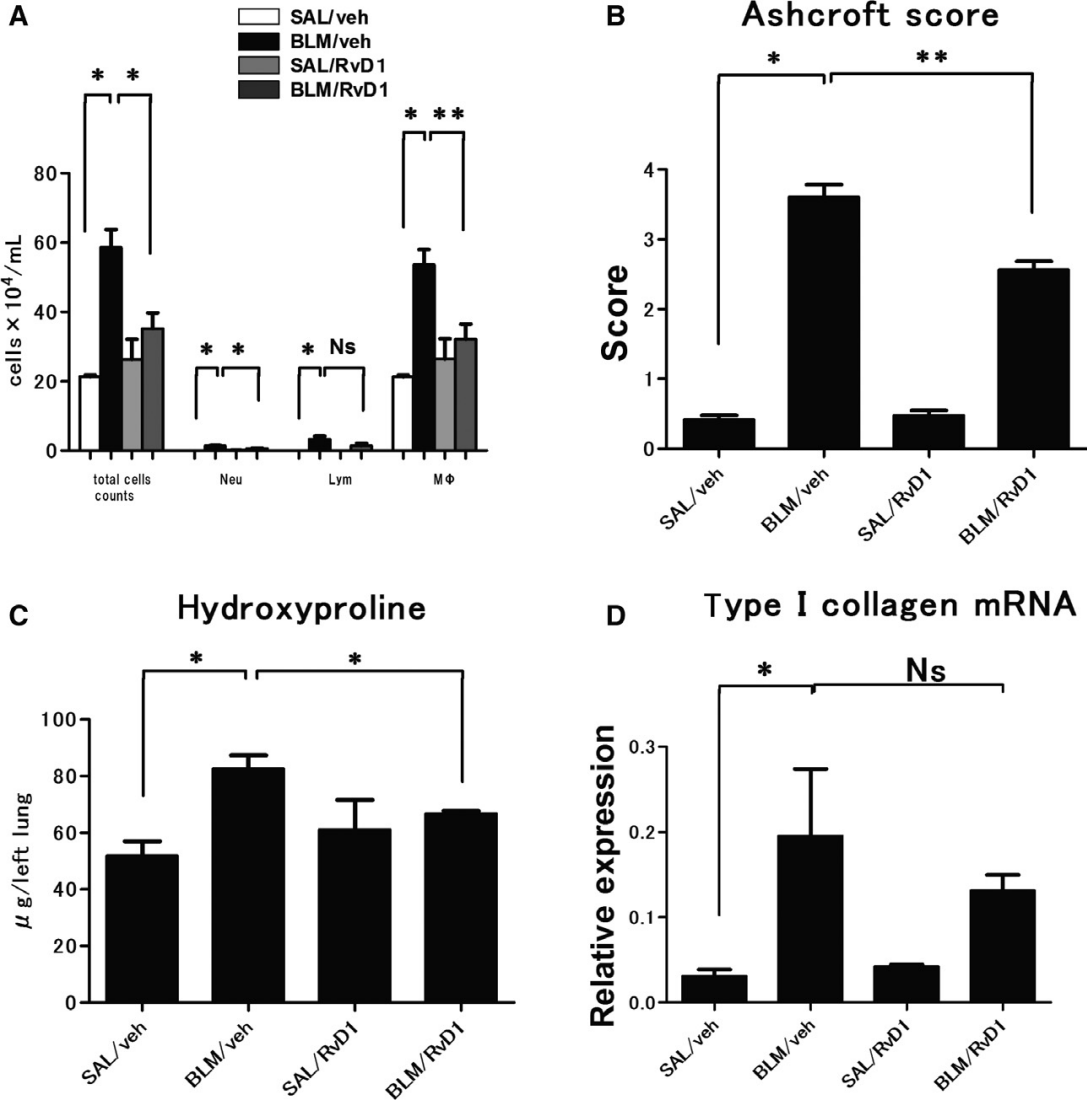
The assessment of chronic inflammation and fibrotic change in bleomycin (BLM)‐treated mice in the second protocol. (A) cell counts in BAL in each group at day 28 after vehicle or 17(R)‐RvD1 (here abbreviated as RvD1) administration from day 21 to day 25. Data are expressed as means ± SEM of five mice per group (*n* = 5). (B) Ashcroft score, pathological assessment of the grade of pulmonary fibrosis at day 28 in each group. Data are expressed as means ± SEM of five mice per group (*n* = 5). (C) hydroxyproline contents in lung tissue in each group at day 28. Data are expressed as means ± SEM of five mice per group (*n* = 5). (D) Transcription levels of Type I collagen mRNA in each group at day 28 were measured by qRT‐PCR. Data are expressed as means ± SEM of five mice per group (*n* = 5). *P <* *0.05; **P <* *0.01.

**Figure 9:**
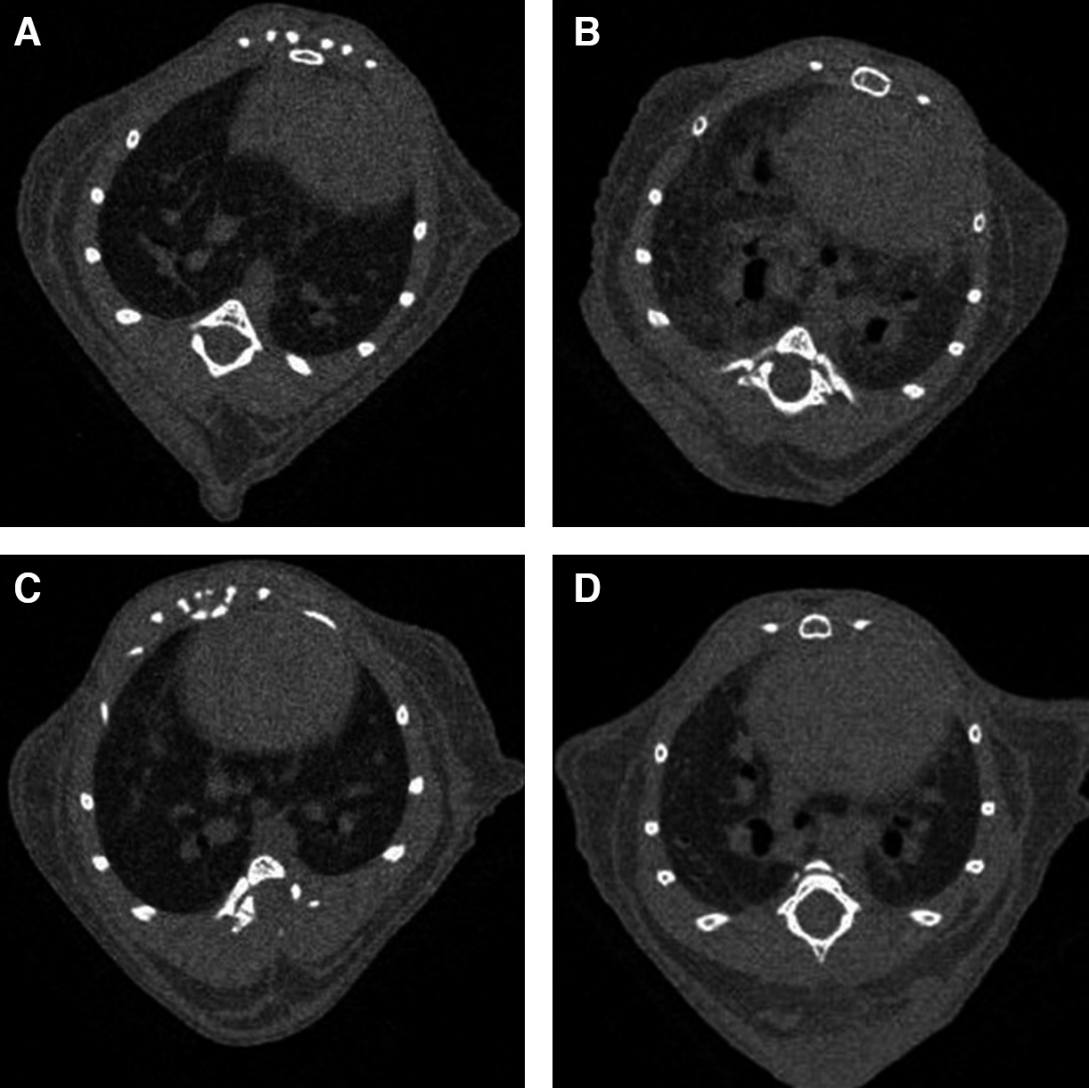
Computed tomography (CT) images of lung tissue. Density of mouse lung in the second protocol was analyzed as visualized CT image at day 28 after vehicle or 17(R)‐RvD1 administration from day 21 to day 25. The increase in lung density may indicate more extensive fibrosis and chronic inflammation. (A) SAL/veh treatment. (B) bleomycin (BLM)/veh treatment. (C) SAL/RvD1 treatment. (D) BLM/RvD1 treatment.

**Figure 10:**
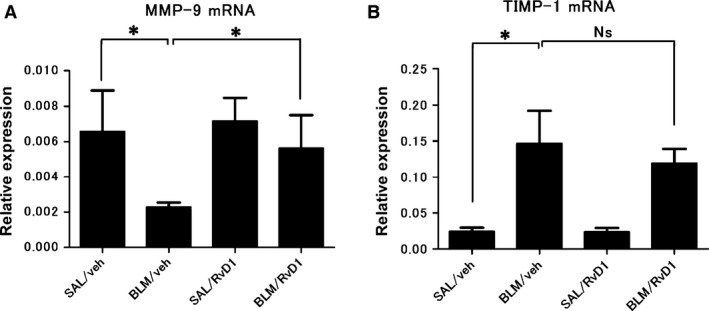
Expression of MMP‐9 and TIMP‐1 mRNA. The gene expressions of MMP‐9 and TIMP‐1 in lung tissue at day 28 were measured to resolve the reduction of fibrotic change in mouse lung after 17(R)‐RvD1 administration during fibrotic stage. Relative levels of expression of MMP‐9 (A) and TIMP‐1 (B) mRNAs were measured by qRT‐PCR. Data are expressed as means ± SEM of five mice per group (*n* = 5). *P < 0.05.

In the present study, we evaluated the effects of 17(R)‐RvD1 by two protocols. In the first protocol, 17(R)‐RvD1 was administered at day 0 simultaneously with BLM and, in the second protocol, the lipid mediator was provided from day 21 for five consecutive days after BLM administration (Fig. 1). Thus, we can know the effects of 17(R)‐RvD1 on the onset of fibrosis in the first protocol and on the established fibrosis in the second protocol. We first examined the effects of 17(R)‐RvD1 by the first protocol. It is noted that the experiments of Figures 2, 3, 4, 5, 6 are carried out by the first protocol and those of Figures 7, 8, 9, 10 are by the second protocol.

To evaluate the anti‐inflammatory effect of 17R‐RvD1 on BLM‐induced lung injury, mice were implanted with micro‐osmotic pumps containing either BLM or SAL, and subsequently injected with 17(R)‐RvD1 or vehicle. An active inflammatory response is characterized by neutrophils, lymphocytes, and macrophages infiltrating the interstitium and the alveolar spaces. Therefore, the total number of such cells and the distribution of immune cell types in BALF were assessed at day 0, 7, 14, and 28 after BLM administration, and the expression of IL‐1*β* was assessed at day 14 in our experimental model (Fig. 1). At day 14 in the first protocol, the numbers of neutrophils, macrophages, lymphocytes, and total immune cells were markedly increased in BALF from BLM‐treated mice relative to the numbers in BALF from saline‐treated controls (Fig. 2A). At day 7, during onset of the acute inflammatory phase, neutrophils in BAL increased after treatment with BLM (data not shown). However, compared with injection with vehicle only, injection with 17(R)‐RvD1 after BLM treatment resulted in no statistical difference in the number of neutrophils in BALF (Fig. 2B) and also in IL‐1*β* expression (data not shown) at day 7. At days 14 and 28, there were statistically fewer neutrophils in BALF from mice treated with BLM and injected with 17(R)‐RvD1 (i.e., mice in the BLM/RvD1 group) versus in BALF from mice treated with BLM and injected with vehicle only (i.e., mice in the BLM/veh group) (Fig. 2B). This result was consistent with the decrease in the numbers of macrophages in BALF from BLM/RvD1 group mice versus from BLM/veh group mice at day 14 (Fig. 2A).

In the experimental mouse model of pulmonary fibrosis utilized in this study, BLM induces pneumonitis, which leads to fibrotic changes resulting from increased IL‐1*β* and TGF‐*β*1 production (Sleijfer 2001). IL‐1*β* is a potent pro‐inflammatory cytokine that induces the progressive conversion of acute tissue injury into fibrosis (Kolb et al. 2001)**,** and is produced by leukocytes such as neutrophils, macrophages, and lymphocytes. In our model, the expression of IL‐1*β* was significantly lower in BLM/RvD1 group mice than in BLM/veh group mice (Fig. 2C).

### The anti‐fibrotic effect of 17(R)‐RvD1 in the BLM mouse model

To evaluate the anti‐fibrotic effects of treatment with 17(R)‐RvD1 on BLM‐induced lung fibrosis, lung collagen content, histopathology, levels of type I collagen mRNA, and computer tomography images were assessed at day 28, when lung fibrosis was expected to be the most pronounced. Total collagen content in the lung was assayed as a measure of the extent of fibrosis. At day 28, tissue sections from mice in the BLM/veh group displayed multifocal areas of interstitial fibrosis and collapsed alveoli, along with some areas of inflammation with cell infiltrates, as demonstrated by H&E staining (Fig. 3B). There was much less disruption of the lung architecture when mice were injected with 17(R)‐RvD1 following BLM administration (i.e., in the BLM/RvD1 group of mice) (Fig. 3D)**.** Lungs from mice injected with 17(R)‐RvD1 following saline administration (i.e., the SAL/RvD1 group of mice) (Fig. 3C) were indistinguishable from lungs from mice injected with vehicle following saline administration (i.e., the SAL/veh group of mice) (Fig. 3A). Staining collagen with Masson's trichrome revealed that increased amounts of extracellular matrix deposition were observed in the lung sections from mice exposed to BLM (Fig. 3F, H), whereas no fibrotic lesions were observed in stained histology sections from mice treated with saline (Fig. 3E, G). The histology of the lung sections from mice in the BLM/RvD1 group exhibited less collagen deposition (Fig. 3H) than that of lung sections from mice in the BLM/veh group (Fig. 3F), indicating that the 17(R)‐RvD1 injections reduced the level of collagen deposition and associated fibrosis.

Next, the Ashcroft scale was applied to the quantitative histological analysis of the fibrotic changes induced by BLM. The mean score for all sections from mice in the BLM/RvD1 group was significantly lower than that for all sections from mice in the BLM/veh group (Fig. 3I), indicating that injections with 17(R)‐RvD1 reduced the severity of fibrosis.

Hydroxyproline content of lung tissues, a marker for collagen deposition, was also assessed, as representative of the degree of fibrosis. Injections with 17(R)‐RvD1 quantifiably reduced the hydroxyproline content in sections from mice exposed to BLM (Fig. 3J, BLM/RvD1 vs. BLM/veh). Type I collagen is the major fibrous collagen synthesized by wound fibroblasts during the repair process. The primary source of expression of type I collagen in a region of damaged tissue is the myofibroblast, so high levels of the corresponding mRNA indicates the presence of myofibroblasts, which, in turn, indicates progressive fibrosis. Uninjured lungs expressed little of this mRNA, whereas the level of this mRNA was increased in mice following administration of BLM. However, relative to the expression of this mRNA in the lungs from mice in the BLM/veh group, there was significantly reduced expression in the lungs of mice treated with 17(R)‐RvD1 after BLM administration (i.e., mice in the BLM/RvD1 group) (Fig. 3K).

### CT of mouse lungs

In addition to histopathological morphologic alterations, the assessment of lung density by CT examination enabled us to quantify the scale of pulmonary fibrosis, since an increase in lung density could indicate greater inflammation and fibrosis. The detection of the extent of fibrotic disease progression by CT examination correlates well with the extent as determined by histology and clinical impairment (Abdollahi et al. 2005). Therefore, we analyzed all samples in each group in this study (data not shown). Figure 4 displayed representative images of radiological features in the lungs from mice in each group. Exposure to BLM (followed by injections with vehicle only) gave rise to substantial fibrotic changes, as demonstrated by higher density areas and signs of traction bronchiectasis (Fig. 4B). Injections of 17(R)‐RvD1 reduced the effects of BLM (Fig. 4D vs. Fig. 4B). Injections of 17(R)‐RvD1 after administration of saline produced only minor changes, when compared with (control) injections of vehicle after saline administration (Fig. 4C vs. Fig. 4A). Injection with 17(R)‐RvD1 was visibly able to attenuate the typical radiologically detectable features of the inflammatory response, and of fibrotic changes, in lungs from mice treated BLM.

### Effect of 17(R)‐RvD1 on the process of fibrosis

TGF‐*β*1 and CTGF are components of TGF‐*β* signaling that are closely associated with fibrotic changes in multiple organs. Therefore, we utilized qRT‐PCR to explore the expression of TGF‐*β*1 and CTGF, respectively, in mouse lungs. Levels of expression of TGF‐*β*1 (Fig. 5A) and CTGF (Fig. 5B) at day 14 after BLM administration were significantly decreased in the lungs of mice injected with 17(R)‐RvD1 (i.e., BLM/RvD1 group mice). These results indicated that 17(R)‐RvD1 administration during the inflammatory phase reduced the extent of fibrosis by inactivating TGF‐*β* signaling.

### Effect of Boc‐PLPLP, the ALX/FPR2 receptor antagonist

G protein‐coupled receptors, lipoxin A4 receptor/formyl peptide receptor 2 (ALX/FPR2) and G protein‐coupled receptor 32 (GPR32), have recently been identified as receptors for RvD1 (Krishnamoorthy et al. 2010). To explore the receptor for RvD1 involved in our system, we used Boc‐PLPLP, an antagonist for ALX/FPR2. As shown in Figure 6, Boc‐PLPLP reversed the RvD1‐induced inhibitory action on the infiltration of neutrophils and macrophages in response to BLM. These results suggest that RvD1‐induced infiltration of inflammatory cells in lung tissues and hence the subsequent progression of fibrotic response is mediated by ALX/FPR2.

### The anti‐fibrotic effects of 17(R)‐RvD1 during established fibrotic stage

We next focused on the anti‐fibrotic effects of 17(R)‐RvD1 administration during established fibrotic stage using the second protocol (protocol 2 in Fig. 1). In this protocol, 17(R)‐RvD1 was administered to BLM‐treated mice from day 21 for five consecutive days during the fibrotic stage. At day 28, we measured several inflammatory and fibrotic markers as follows. As shown in Figure 7, we investigated the fibrotic change in BLM‐injected mouse lung after 17(R)‐RvD1 administration. A sample of BLM/RvD1 group (Fig. 7D, H) showed less BLM‐induced damaged area and ECM accumulation than BLM/veh group (Fig. 7C, F). As shown in Figure 8, 17 (R)‐RvD1 significantly reduced the number of inflammatory cells in BAL (Fig. 8A). This result indicates 17(R)‐RvD1 has anti‐inflammatory ability in fibrotic stage as well as non‐fibrotic stage. In addition, 17(R)‐RvD1 significantly decreased Ashcroft score (Fig. 7B) and the hydroxyproline content (Fig. 7C). These results suggest that 17(R)‐RvD1 attenuated fibrosis and the accumulation of ECM with damage to lung structure. However, there was no significant difference in type*Ι*collagen mRNA at day 28 between BLM/veh group and BLM/RvD1 group, although 17(R)‐RvD1 tends to decrease the mRNA expression (Fig. 8D).

CT image is also available for assessment of chronic inflammation and fibrotic change as well as histopathology. We examined all samples of each group in this protocol (data not shown). And we discovered the characteristic of CT image in each group. Figure 9 shows one representative sample of each group. In CT image, the sample of BLM/RvD1 group showed the lower density than BLM/veh group. This result suggests that 17(R)‐RvD1 may lead BLM‐induced chronic inflammation and fibrosis to normal lung architecture.

As shown in Figure 10, we further investigated the mechanisms of reduction of fibrotic change. We examined the gene expressions of MMP‐9 and TIMP‐1 in lung tissue at day 28 to find more detailed anti‐fibrotic effect of 17(R)‐RvD1. In second protocol, BLM significantly decreased gene expression of MMP‐9 and, on the contrary, increased TIMP‐1 expression at day 28 (Fig. 10). In the RvD1‐treated animals, BLM did not appreciably affect MMP‐9 expression, whereas it again increased TIMP‐1 expression (Fig. 10). These results suggest that 17(R)‐RvD1 provides pulmonary restoration via increasing MMP‐9 mRNA close to the normal level.

## Discussion

In this study, we provide what is, to our knowledge, the first evidence of an anti‐fibrotic effect by 17(R)‐RvD1, a known anti‐inflammatory lipid mediator derived from the *ω*‐3 polyunsaturated fatty acid DHA, in a mouse model of BLM‐induced pulmonary fibrosis. Administration of 17(R)‐RvD1 inhibited BLM‐mediated stimulation of pro‐inflammatory cytokines, suppressed the recruitment of neutrophils into the alveolar spaces, and reduced expression of pro‐fibrotic cytokines and deposition of ECM, thereby ameliorating lung fibrosis. Although a previous study from Liao et al. (2012) revealed that RvD1 significantly and rapidly reduces the numbers of neutrophils in BALF, inhibits NF‐*κ*B activation, and attenuates inflammation in lung tissue, the mechanisms of its anti‐fibrotic effects remained unclear until now.

Based on our findings, 17(R)‐RvD1 attenuated the development of BLM‐induced pulmonary fibrosis in mice possibly through the downregulation of transcription of IL‐1*β* mRNA in mouse lung, and through the reduction in infiltration of neutrophils into bronchoalveolar tissues and BALF. Neutrophilia is a common feature of pulmonary fibrosis, and BALF neutrophilia has been identified as a common finding in IPF patients (Hunninghake et al. 1981). Increased BALF neutrophil percentage is an independent predictor of early mortality among patients with IPF (Kinder et al. 2008), which indicates that neutrophils play pivotal roles in IPF. Sustained neutrophil accumulation in the alveolar space and neutrophil‐mediated injury to the alveolar wall are believed to play a role in interstitial fibrosis and abnormal lung repair. Neutrophils first migrate into tissues to participate in host defense, and then, if the cause of the inflammation is successfully removed, these tissues return to homeostasis. As part of general resolution, tissue‐level resolution programs are actively initiated, and the number of tissue‐associated neutrophils is reduced (Serhan et al. 2007). However, chronic or excessive inflammation can result from insufficient, delayed, or failed resolution, and can lead to several common respiratory diseases (Haworth and Levy 2007). Therefore, the proper promotion of resolution of the inflammatory response is important for the attenuation of lung fibrosis. It has been previously shown that 17(R)‐RvD1 possesses anti‐inflammatory and pro‐resolving abilities and can mediate resolution of inflammation (Sun et al. 2007). Our findings reinforce the notion that 17(R)‐RvD1 has anti‐inflammatory functions. In addition, our data suggest that 17(R)‐RvD1 exerts an anti‐inflammatory and pro‐resolving effect on pulmonary immune cells such as neutrophils, and thereby contributes to the attenuation of chronic inflammation and fibrotic changes caused by BLM exposure.

The specific receptors that bind RvD1 were recently identified as the ALX/FPR2 and GPR32 (Krishnamoorthy et al. 2010), which are found on human neutrophils, monocytes, and macrophages. Pro‐inflammatory stimuli mobilize ALX/FPR2 receptors to the neutrophil surface (Norling et al. 2012). RvD1 activates these receptors, reducing excessive neutrophil infiltration into inflamed tissues and decreasing neutrophil activation, but promoting phagocytosis in the clearance of apoptotic cells (Spite and Serhan 2010). Thus, neutrophil recruitment, consisting of neutrophil capture, rolling, and adhesion, is likely directly attenuated by binding of RvD1. Blockage by Boc‐PLPLP, an antagonist of ALX/FPR2, on the RvD1‐induced action, supports the hypothesis that 17(R)‐RvD1 executes its pro‐resolving actions within lung tissues via its interactions with ALX/FPR2.

As stated above, in our model, 17(R)‐RvD1 protected against BLM‐induced pulmonary fibrosis by downregulating transcription of mRNAs encoding IL‐1*β* in the lungs. IL‐1*β* is one of a family of pro‐inflammatory cytokines thought to be involved in many acute and chronic diseases. The pro‐IL‐1*β* form is constitutively expressed in pulmonary immune cells, such as neutrophils, monocytes, and macrophages. Moreover, previous studies have shown that human alveolar macrophages produce IL‐1*β* in response to BLM (Scheule et al. 1992). The expression of IL‐1*β* was lower in lungs from BLM/RvD1 group mice than in lungs from mice in the BLM/veh group. This finding suggests that 17(R)‐RvD1 decreased the production of this pro‐inflammatory cytokine in pulmonary cells and contributed to the promotion of the resolution of the inflammatory response and the return to a non‐inflamed state.

IL‐1*β* induces the release of active TGF‐*β*1, and triggers expression of TGF‐*β*1 mRNA by activating the NF‐*κ*B pathway. Furthermore, IL‐1*β* is linked to fibrosis through TGF‐*β* signaling, although not directly through increased inflammation (Lee et al. 2006; Gupte et al. 2009). Since TGF‐*β*1 induces chemotaxis and stimulates the activity of cells that produce ECM, this cytokine is a very potent inducer of tissue fibrosis (Yaekashiwa et al. 1997). TGF‐*β*1 plays an important role in the pathogenesis of lung fibrosis through its strong extracellular matrix‐inducing effect (Willis and Borok 2007) and also promotes differentiation of fibroblasts into myofibroblasts, which triggers enhanced expression of alpha‐smooth muscle actin and type I collagen, and suppresses IL‐1*β*‐induced apoptosis (Serini et al. 1998; Phan 2002). Like TGF‐*β*1, CTGF is also implicated in various fibrotic disorders and is induced in fibroblasts after activation by TGF‐*β*1. Furthermore, CTGF promotes cell adhesion and migration in a wide variety of cell types, as well as collagen matrix contraction in fibroblasts (Babic et al. 1999; Shi‐Wen et al. 2000; Chen et al. 2001). Treatment with 17(R)‐RvD1 counteracted the increase in TGF‐*β*1 and CTGF expression stimulated by BLM exposure. Taken together, these results strongly suggest that 17(R)‐RvD1 prevented chronic inflammation, myofibroblast differentiation and ECM accumulation by downregulating the acute inflammatory response through the suppression of TGF‐*β*1 mRNA.

The effects of 17(R)‐RvD1 on alleviating BLM‐mediated fibrosis, on a tissue level, was evident by CT imaging. Although CT imaging in mice has not yet been established as an adequate means to assess the degree of BLM‐induced lung fibrosis, it plays an important role in the diagnosis of IPF in humans. In this study, CT imaging was employed to visualize the degree of BLM‐induced lung fibrosis after 17(R)‐RvD1 administration. We confirmed that a higher density of mouse lung by CT imaging indicated an injured and fibrotic area, so CT imaging may show promise as a major means by which to examine the effects of various agents in mouse models of pulmonary fibrosis. In human with IPF, not only CT images but also quantitative studies on lung compliance and function are important modalities to assess the progression of fibrotic change. However, in mice experiments, it is so difficult to assess the lung compliance and function. A further study about this issue is needed.

Matrix metalloproteinases (MMPs) produced by neutrophils, macrophages, and alveolar epithelium cells have been implicated in the pathogenesis of pulmonary fibrosis. MMP‐9 and TIMP‐1 play an important role in the development of pulmonary fibrosis. The previous study has shown that keeping normal lung architecture partly depends on the proper MMP‐9/TIMP‐1 balance (Wang et al. 2011b). If MMP‐9 is decreased and TIMP‐1 is increased in lung tissue, it promotes ECM accumulation leading to pulmonary fibrosis. BLM decreased MMP‐9 mRNA and increased TIMP‐1 mRNA that caused to accumulate collagen deposition. Our result suggests that 17(R)‐RvD1 administered during the fibrotic stage ameliorates pulmonary fibrosis through restoring MMP‐9 mRNA dramatically.

In the current study, we presented the results of the effect of 17(R)‐RvD1 on expression of mRNA but not protein of TGF*β*, collagen, MMP‐9, and TIMP‐1. We realized that the measurement of protein expression of these cytokines and growth factors would be more meaningful to elucidate the process underlying the restoration of pulmonary fibrosis. Nevertheless, we believe that our present results even without their protein expression are very important from the point of view as therapeutic drugs against fibrosis. Further experiments are surely necessary to fully understand the 17(R)‐RvD1 actions on pulmonary fibrosis.

Although we have not yet examined completely whether 17(R)‐RvD1 can restore a fully fibrotic lung to normal, we were able to demonstrate that 17(R)‐RvD1 possessed not only anti‐inflammatory, but also anti‐fibrotic capabilities that served a protective function and the decrease of collagen deposition. Moreover, in the context of drug development, it is important to note that 17(R)‐RvD1 is an endogenous, physiologically active molecule that resists rapid inactivation; therefore, exogenously generated 17(R)‐RvD1 is expected to be relatively safe and highly effective in preventing onset of IPF. Interestingly, we found that 17(R)‐RvD1 may have a potential to provide pulmonary restoration. 17(R)‐RvD1 administered even during fibrotic stage reduced chronic inflammation and fibrotic change: the results of BALF, hydroxyproline content, Ashcroft score, pathohistology, CT image were highly similar to those in the first protocol. A greater understanding of anti‐inflammatory, anti‐fibrotic, and pro‐resolution molecules promoted by studies such as this one, is providing new insights and opportunities to design therapeutic strategies for pulmonary fibrosis. We hope that these findings will be a step toward discovering new drugs, and developing novel therapeutics, for treating IPF.

## Conflict of Interest

No conflicts of interest, financial or otherwise, are declared by the authors.
